# Supplementary material for: Sex-specific effects of cooperative breeding and colonial nesting on prosociality in corvids
Source: eLife. 2020 Oct 20;9:e58139. doi: 10.7554/eLife.58139 (PMC7609055; doi:10.7554/eLife.58139)
Supplement: Figure 2—source data 1. — Given are estimates, standard errors (SE), z-values, sum of AICc weights (SWAICc), and number of models containing the specific factor (NModels) after model averaging. Factors with a sum of AICc weights larger than 0.5 and whose SE of the estimates did not overlap 0 were considered to have a high explanatory degree and are given in bold. Number of individuals: N = 51. [file elife-58139-fig2-data1.docx]

| **Parameter** | **Estimate** | **SE** | **Z** | **SW_AICc_** | **N_Models_** |
| --- | --- | --- | --- | --- | --- |
| (Intercept) | 6.188 | 3.888 | 1.557 | - | - |
| **Cooperation (yes)** | **10.024** | **4.499** | **2.168** | **0.67** | **1** |
| Nesting (territorial) | 4.150 | 4.283 | 0.944 | 1.00 | 2 |
| **Sex (male)** | **17.432** | **5.881** | **2.903** | **1.00** | **2** |
| **Cooperation (yes) x Sex (male)** | **-16.057** | **6.861** | **2.277** | **0.67** | **1** |
| **Nesting (territorial) x Sex (male)** | **-19.763** | **6.319** | **3.048** | **1.00** | **2** |
