# Supplementary material for: Sex-specific effects of cooperative breeding and colonial nesting on prosociality in corvids
Source: eLife. 2020 Oct 20;9:e58139. doi: 10.7554/eLife.58139 (PMC7609055; doi:10.7554/eLife.58139)
Supplement: Figure 2—source data 2. — Given are estimates, standard errors (SE), z-values, sum of AICc weights (SWAICc), and number of models containing the specific factor (NModels) after model averaging. Factors with a sum of AICc weights larger than 0.5 and whose SE of the estimates did not overlap 0 were considered to have a high explanatory degree and are given in bold. [file elife-58139-fig2-data2.docx]

| **TABLE A) Females (N=26)** | | | | | |
| --- | --- | --- | --- | --- | --- |
| **Parameter** | **Estimate** | **SE** | **Z** | **SW_AICc_** | **N_Models_** |
| (Intercept) | 6.854 | 3.105 | 2.111 | - | - |
| **Cooperation (yes)** | **9.686** | **4.427** | **2.076** | **1.00** | **2** |
| Nesting (territorial) | 4.469 | 4.136 | 1.024 | 0.32 | 1 |
| **TABLE B) Males (N=25)** | | | | | |
| **Parameter** | **Estimate** | **SE** | **Z** | **SW_AICc_** | **N_Models_** |
| (Intercept) | 22.798 | 3.784 | 5.729 | - | - |
| Cooperation (yes) | -6.032 | 5.266 | 1.083 | 0.33 | 1 |
| **Nesting (territorial)** | **-15.066** | **4.528** | **3.154** | **1.00** | **2** |
